# Cone Beam CT Assessment of Bone Width of Upper and Lower Jaws for Dental Implant Placement: An Iraqi Study

**DOI:** 10.1155/2023/4472154

**Published:** 2023-04-13

**Authors:** Aseel S. Khazaal Al-Jaboori, Nuhad A. Hassan

**Affiliations:** ^1^Department of Prosthetic Dentistry, College of Dentistry, AL-Mustansiriyah University, Baghdad, Iraq; ^2^Department of Oral Medicine, College of Dentistry, AL-Mustansiriyah University, Baghdad, Iraq

## Abstract

**Background:**

Implantology focuses on the measurement of bone thickness in both the lower and upper jaws. This study aimed to measure and compare alveolar bone thickness of the upper and lower jaws at single edentate sites and cortical bone thickness of their mesial and distal dentate sites.

**Methods:**

Thickness of alveolar bone thickness was measured in 80 upper and 80 lower implant edentate sites and that of buccal and lingual cortical plates of their mesial and distal dentate sites using Cone beam CT. The bone thickness of the edentulous sites was recorded at 3 points (crestal bone, five mm from the crest, and ten mm from the crest), while the bone thickness of the dentate sites was determined at four points (crestal bone, midroot bone, mid of the alveolar bone housing, and apical portion).

**Results:**

An increased amount of bone was measured from the crest to the apical portion of the dentate sites on the buccal and lingual sides of both jaws with a highly significant difference detected among all the tested points (*P* <  0.0001). No statistical difference was detected between the means of buccal bone width at the first 3 points, except at point 4 (the apical portion), where the mean of the lower jaw (3.35 ± 0.54) was significantly larger than that of the upper jaw (3.17 ± 0.55) (*P* = 0.04). Bone width measured in the edentulous sites showed a gradual increase from the crest to the apical portions in both jaws.

**Conclusion:**

Bone thickness at the coronal levels is low and susceptible to resorption compared to the apical portions regardless of the dentate state.

## 1. Introduction

When a tooth is lost, bone and soft tissue resorption is unavoidable, which may result in a significant reduction in residual bone volume and may jeopardize implant placement that is essential for an optimum restoration. Therefore, paying close attention to the primary bone structure is critical when planning for dental implant therapy, and extra caution is advised in cases where the alveolar bone thickness is thin [1]. Patients with sufficient cortical bone thickness surrounding a cancellous bone were shown to be the best candidates for implant therapy [2].

Implants inserted in cortical bone needed more torque for their removal than the cancellous bone implants [3]. Moreover, the thickness of the cortical bone had a bigger influence on the initial stability of the implants compared to the implant length [4].

Implantology focuses on the measurement of bone thickness in both the lower and upper jaws. Analysis of the anatomical pattern of the bone has a good role in the surgical preparation of the implantation process and the results of dental implant reconstruction [5, 6]. Even though periapical radiographs and orthopantomogram can help in determining the socket dimensions, they only provide a two-dimensional picture. They are not 100 percent accurate and reliable as a diagnostic tool because they have disadvantages such as projection geometry, superimpositions, and lack the third dimension of bone depth [7]. Computed tomography (CT) and cone beam CT (CBCT) were developed later to allow the surgeon to visualize the implant site in 3-dimensions and the relative skeletal anatomy such as the mandibular nerve and maxillary sinus [7, 8]. CBCT is employed widely in dentistry for diagnosis and treatment planning because of its short scanning time, low dose, and good resolution compared to CT scanning [9].

In a study carried out by Lupi et al. [10], geometric distortion of CBCT software tools was assessed by comparing CBCT-generated panoramic angle between the axis of 2nd and 3rd molars to that of 3D CBCT angle. They found that geometric distortion is the only source of distortion in CBCT-generated panoramic images that may influence surgical planning of impacted 3^rd^ molar.

Previous researchers recorded perforation risks in the lingual plate of the posterior part of the mandible during implant placement. Thus, they recommended a preoperative examination by CBCT before implant placement as a protective indicator [11, 12].

Bone thickness was evaluated previously in an Iraqi study carried out by Al Tekreeti et al. [13] who used occlusal films to evaluate the buccolingual width of mandibular molar areas for 30 subjects in relation to the dentate state. Whereas, another previous study focused on the bone width of both jaws using advanced technology [14]. However, it has the limitation that the site dimensions of the lower and upper jaws were combined. The aim of this study was to analyze bone width buccally and lingually of posterior implant sites in relation to dentate state and jaw site using CBCT for an Iraqi sample.

## 2. Materials and Methods

Individuals with missing molars or premolars were analyzed. The data were separated by regions equally into 80 upper arch and 80 lower arch sites of the patients (aged between 22 and 52 years). According to the Declaration of Helsinki as well as the World Medical Association, the Scientific Committee of the Department of Oral Medicine approved the local ethical standards (PROTOCOL 14 FEBRUARY 2021). The consent of all participating patients was obtained. Patients were seeking CBCT for different dental treatments at the Specialized Center of Dentistry in Al-Sadar City from February 2021 to September 2021.

Patients with missing teeth and healthy periodontal tissue were included in this study, excluding any cases with periapical lesions or a history of surgery. The patient information was collected by Kodak 9500, Care Stream, France. Exposure time: 10.8 s, 90 KV, 10 mA, voxel size: 300 *μ*m, and FOV: 8 × 8 cm.

The mean values were recorded for all the data, which were estimated and repeated after one week by the same radiologist. The two readings were nearly identical, but a paired *t*-test of 10 random CBCT scans revealed no significant differences, indicating intraobserver reliability (Table 1). In the statistics, the mean of each of the two measurements was used. To decrease artifacts caused by metallic fillings such as streaking, the teeth should be put aside from the central field of view (FOV) [15].

The scans evaluated the teeth directly on mesial and distal areas next to the edentulous regions. If the first molar was missing, the second premolar and second molar were measured instead. The image analysis of the scans was carried out using imaging software (CS 3D Imaging v3.5.7). As a reference, a line was vertically drawn throughout the length of the target tooth through the sagittal plane, and another line was drawn horizontally from the buccal to palatal/lingual surfaces at the location of the ridges. Bone thickness of buccal and lingual walls was measured in dentate regions along the long axis of the root vertically. The measurements were done at four different points along the bone walls of the chosen teeth in coronal plane as follows [14]:

1. Point 1 (P1)-Crestal bone thickness
2. Point 2 (P2)-Midroot bone thickness
3. Point 3 (P3)-Middle of the alveolar bone housing
4. Point 4 (P4)-Apical portion of the tooth bone thickness

**Figure 1:**
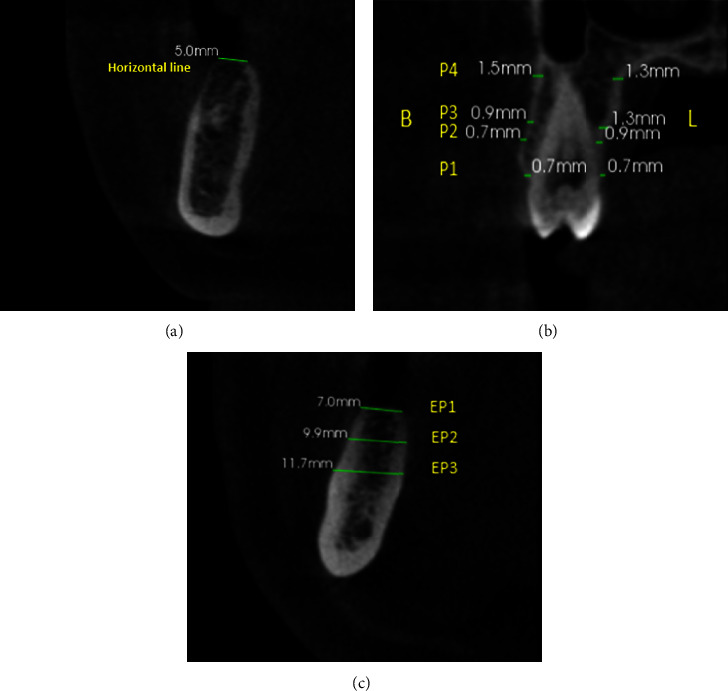
(a) Horizontal line: a line was drawn horizontally from the buccal to palatal/lingual surfaces at the location of the ridges. (b) Bone thickness of buccal and palatal/lingual walls was measured vertically to the root in dentate regions through coronal view at four points: crestal bone thickness (P1), midroot bone thickness (P2), middle of the alveolar bone housing (P3), and apical portion of the tooth bone thickness (P4). (c) Alveolar ridge thickness in edentulous regions at three points: crestal ridge thickness (EP1), second point 5 mm from crestal (EP2), and third point 10 mm from crestal (EP3).

In edentulous areas, the thickness of the alveolar bone was measured through the mesiodistal center at 3 different points (Figures 1(a)–1(c)) as follows:

1. EP1: Crest bone thickness
2. EP2: Bone thickness at 5 mm from EP1
3. EP3: Bone thickness at 10 mm from EP1

## 3. Results

This study evaluated the bone width of 160 sites for patients demanding a single posterior tooth implant (age range 22–52 years), comprising 80 upper jaw sites and 80 lower jaw sites. Bone width of mesial and distal located dentate sites was measured at 4 points and that of the edentulous sites at 3 points.

The difference in bone width between the points of each dentate and edentulous site was statistically analyzed using a Kruskal–Wallis one-way analysis of variance. The independent sample *t*-test was used to test the statistical difference between the upper and lower site groups and between buccal and lingual sides at each point.

As shown in Table 2, an increased amount of bone was measured from the crest (P1) to the apical portion (P4) of the dentate sites on the buccal and lingual sides of both jaws with a highly significant difference detected among all point groups (*P* *<* 0.001, Kruskal–Wallis test).

The mean bone width of upper sites was increased from P1 (1.14 ± 0.15 mm) to P4 (3.17 ± 0.55 mm) on the buccal side, and similarly increased on the lingual side from 1.18 ± 0.18 mm at P1 to 3.27 ± 0.61 mm at P4.

A similar trend was recorded for the lower jaw groups when the width of the cortical bone was raised from the alveolar crest to the apical part of the bone (1.16 ± 0.15 mm to 3.35 ± 0.54 mm buccally and 1.20 ± 0.19 mm to 3.44 ± 0.59 mm lingually).

Regarding the differences between the width of the upper and lower jaws in the dentate sites (Table 3), no statistical difference was detected between the means of buccal bone width at the first 3 levels, except at point 4 (apical portion of the bone), where the mean of the lower jaw (3.35 ± 0.54 mm) was significantly larger than that of the upper jaw (3.17 ± 0.55 mm) (*P* = 0.04). While the lingual bone expressed a nonstatistically significant difference between the upper and lower sites for all points with *P* > 0.05.

When comparing bone thickness between the buccal and lingual sides of the dentate sites, no statistical difference was detected in both upper and lower jaw groups at all points (*P* > 0.05).

Alveolar bone width measured in the edentulous sites of upper and lower jaws (Table 4) showed a gradual increase from P 1 to P 3 in both jaws. In the upper sites, crestal bone width was 4.0 ± 0.47 mm and increased to 9.61 ± 0.75 mm (P 3). Likewise, lower bone width increased from 4.09 ± 0.50 mm at P 1 to 9.75 ± 0.60 mm at P 3. The differences in mean values were highly significant across the 3 levels, as indicated by *P* < 0.001 in both jaws (Kruskal–Wallis test).

At each point of the edentulous sites (Table 4), no significant difference was revealed between bone width values of the upper and lower jaws (*P* > 0.05).

## 4. Discussion

Dental implant therapy is frequently used to replace missing teeth. To achieve optimal functional and aesthetic restoration after implant treatment, it is of great importance to have sufficient alveolar bone volume and mesiodistal dimensions in the implant site [16]. Periapical pathology, periodontal diseases, and bone damage or trauma that happened during tooth extraction can all cause alveolar bone loss prior to tooth extraction. Hence, gathering information about contour changes caused by the bone atrophy and remodeling at the extraction sites is essential to perform a highly reproducible presurgical plan [17]. Bone volume and dimensions have been evaluated using different imaging methods. However, the image magnification and distortion of old imaging techniques were resolved by the use of CBCT. The accuracy of linear measurements was increased in CBCT with less mean error (0.1–0.20 mm). Panoramic distortion may show a high percent (20%) compared to CBCT [16–20]. While, the CT has 3–10 times higher radiation exposure than CBCT [18]. Adjustment of CBCT exposure parameters and limitation of the field of view decreased the radiation dose. The role of CBCT starts from the preoperative examination of implant treatment procedures such as anatomical analysis, site augmentation by grafts, and image manipulation by computer to postoperative review for any injury to vital structures [19].

Few CBCT studies have examined the impact of bone thickness on implant treatment planning and final outcomes [14, 20], and none has compared between bone thickness of upper and lower jaws. In the past study that was carried out by Merrot et al. [21] of 67 edentate and 43 dentate old age patients, upper and lower bone width estimation were combined, and the study did not differentiate between them as compared to this work.

In the current study, bone width was evaluated at edentulous sites and the mesial and distal dentate sites at four points (from crest to apex across the whole root length). It is known that the coronal part of the alveolar bone has a stronger impact on the ultimate results of implant restorations including function and aesthetics [22]. However, the apical extent of bone changes after resorption has yet to be determined, and such information would allow dentists to evaluate these structures in implant therapy.

This study revealed that bone width was increased from point 1 to 4 on both lingual and buccal sides. The difference in bone width recorded was highly significant among all point groups in both jaws. Another finding of the present analysis was that no difference was detected in bone thickness between mean values of point 1 (crest) and 2 (midroot) in both buccal and lingual sides, while there was a significant increase in bone width from point 3 onwards. This fact indicates that crest and midroot levels of alveolar bone would have more tendencies to resorption post-tooth extraction than apical and mid alveolar bone housing parts.

In the results revealed by Cassetta et al. [23], there was a drop in alveolar bone thickness from the base to the crest, which was in line with the findings of this work. The same result was found by Kolte et al. [14] in 2020, which conducted a study for 100 individuals using CBCT and found a steadily decreasing in thickness of bone from apical to crest for both lingual and buccal sides. Moreover, this study was a close match to what was found in an old study that showed bone thickness on the buccal side usually decreases from apical to coronal parts [24].

Interestingly, the present study was consistent with the range of thickness published by Vera et al. [25] during the evaluation of upper first premolars, who indicated that the preservation of tissue structures and implant position needed a small thickness of alveolar bone buccally (1 to 2 mm) after tooth extraction. This was also confirmed in the literature where a minimum bone thickness of 2 mm was recommended to minimize complicated aesthetic outcomes and bone reduction in the future [26, 27]. Moreover, a buccal plate of at least 2 mm was suggested by Grunder [28] to avoid soft and hard tissue recession for proper immediate placement of the implants with successful long-term outcomes. When the minimal crest width is not available for implant insertion, the surgeon can perform local bone augmentation. To reduce buccal plate resorption and when the demand for aesthetics is high, bone substitutes were applied to have a low rate of resorption in order to fill the space between the buccal bone wall and the implant [29].

This result is as well compatible with a specific study considering the posterior teeth of the mandible and maxilla in which the authors demonstrated an increasing width of the alveolar plate of the buccal side from the crestal to the apical parts and indicated greater bone thickness in the maxillary arch than in the mandibular [30].

In the current study, the values of the lower jaw thickness were greater than the upper jaw, even though the difference was not statistically significant for values of the first three points but was significant for bone width that was measured at the apical portion (P4) of the buccal side (*P* = 0.04) which may offer more cortical bone for primary implant stability. This finding was harmonious with the Porto et al. [31] publication, which conducted a CBCT analyzing study on 422 images and found a smaller buccal thickness of upper teeth than the lower at the apical portion. However, Porto et al. considered the bone thickness from buccal or lingual/palatal cortical bones to the center of the apical foramen so as to simulate the pathway of sinus tract. In our study, we evaluated buccal and lingual cortical bone thickness at 4 points along the tooth root.

According to the study by Farnsworth et al. [32], cortical bone of the lower jaw had a significantly higher thickness than maxillary cortical bone plate measured during investigation of 52 patients by CBCT images analyzing buccal bone width at a point located 4 mm below the crest.

The current result was in contrast with an earlier study which showed a thinner buccal bone in the maxilla than in the mandible at different levels (2–8 mm from the crest) during a study of 48 CT scans [23]. Similar results were recorded by Abbas and Alhuwaizi [33] at the same levels. Baumgaertel and Hans [34] also recorded a thinner buccal bone in the upper jaw as compared to the lower jaw.

Analogous reports documented a thinner alveolar cortical bone in the maxillary jaw compared to that of the mandibular jaw at various levels [14, 15].

It was previously reported that alveolar bone is mainly compact apically and that the buccal side is thinner than palatal side [35]. In contrast, this study indicated no statistical difference between bone width of buccal and lingual regions of the dentate sites in both upper and lower jaw groups and at all the examined points.

Aydin and Bulut [36] measured bone thickness of the posterior teeth and noticed that the lingual side had a smaller bone thickness than the buccal side of the mandible, which was opposed to our result. The difference might be occurred due to the utilizing of different measurement technique where bone measurements were recorded at 3 mm distance from the apical resection level in their work. On the other hand, when Kolte et al. [14] used CBCT to compare bone dimensions recorded in dentate sites, and at the same levels in this study, they found that bone width was significantly greater on the lingual side at all levels. Whereas, in a CT scan study of the mandibular molar region in men, the cortical bone was thicker on the buccal side than on the lingual side, although the difference on the right side was not significant. While in women, cortical bone measurements were higher lingually as compared to those on the buccal side, with no difference between 1st and 2nd molars [37]. Additionally, the authors of the same study reported a gradual increase in width of the cortical buccal bone from anterior areas to posterior areas, and reported a steady decline in width of the cortical lingual bone of the mandible and related this finding to buccolingual inclination of the molars under the action of masticatory muscles and function.

The controversial results found in the literature could be attributed to differences in measurement methods used, sample size, cite of examination, and technical errors due to the anatomical forms of the jaws and individual differences (e.g., tori and undercuts). Age and sex may be other factors that have an influence on the width of the alveolar bone. Therefore, further quantitative studies are needed using larger sample sizes with a focus on sex and age.

Huynh-Ba et al. [38] assessed the immediate insertion of the implants for 93 individuals in the upper jaw and noticed that the lingual bone was thicker than the buccal side, which was not compatible with the current result. This difference in outcomes may be due to the site of measurements; measurements in the current report were taken for cortical plates of upper and lower jaws at different levels, whereas Huynh-Ba et al. included sites from anterior regions of the maxilla and measured thickness of the palatal and buccal walls of tooth sockets at an area apical to the alveolar crest by 1 mm.

Regarding alveolar bone width measured at the edentulous bone sites of this investigation, a gradual increase in bone values was seen from the crest to the apical portion (upper arch; 4.0 ± 0.47 mm to 9.61 ± 0.75 mm and lower arch; 4.09 ± 0.50 mm to 9.75 ± 0.60 mm). The difference in means of bone width values for both jaw groups was highly significant across the three examined points. This result corroborates those of Kolte et al. [14] and Braut et al. [20]. No significant difference was observed in the current study between the mean bone width values of the upper jaws and those of the lower jaws at all the points (*P* > 0.05).

A previous human cadaver study conducted by Katranji et al. [39] revealed an increase in maxillary edentulous bone width of the molar regions from the crest to 3 mm below the crest (7.88 ± 2.26 mm to 8.29 ± 2.57 mm) and similar increase in the mandibular edentulous molar regions (6.02 ± 1.67 mm to 7.31 ± 2.16 mm). One of the most clinical principles that determine the survival rate of endosteal implants is the width of the existing bone. Dental implant of 4 mm diameter usually needs more than 6 mm of bone thickness to guarantee a sufficient blood supply around the implant. The initial crestal bone loss after implant loading is proportional to the initial width of the bone. Therefore, when the width of the edentulous ridge is more than 6 mm, there is a less crestal bone loss than when less bone width is available [2].

The maxillary and mandibular bone thicknesses were measured in this investigation using a high-resolution CBCT system. Some technical characteristics have an impact on the dimensional measures that are acquired by this imaging assessment such as the field of view (FOV), spatial resolution, voxel size, focal point, and the number of basis images [40]. In this study, to reduce the effect of these factors on the linear measurements of bone width, images of CBCT system were determined with submillimeter isotropic voxel (0.3 mm), high resolution, and small FOV. Accordingly, the high-resolution system that was utilized in this CBCT study could be regarded as a reliable instrument in determining bone thickness.

One of the limitations of this examination for the edentulous sites was that the measurements of bone width by CBCT may have been affected by the time duration of tooth loss, tooth replacement with a partial denture, and duration of the denture usage by the patient which might increase bone resorption, probably leading to discrepancies among individuals within the population. Therefore, additional research into these variables is recommended.

## 5. Conclusions

Coronal bone is thinner than apical bone, indicating that coronal bone is more prone to resorption. No variation in bone thickness between the upper and lower jaws was observed. The current research supports the use of CBCT in bone evaluation for implant therapy in order to get a better clinical result.

## Figures and Tables

**Table 1:** Evaluation of intraobserver reliability for variables (mm).

Variable^a^	1^st^ reading	2^nd^ reading	*P* value
	Mean ± SD	Mean ± SD	
P1	1.41 ± 0.27	1.41 ± 0.27	0.44^*∗*^
P2	1.38 ± 0.34	1.37 ± 0.32	0.35^*∗*^
P3	2.4 ± 0.6	2.41 ± 0.59	0.59^*∗*^
P4	3.31 ± 0.87	3.32 ± 0.87	0.59^*∗*^
EP1	4.4 ± 0.39	4.39 ± 0.39	0.10^*∗*^
EP2	7.46 ± 0.58	7.46 ± 0.58	0.59^*∗*^
EP3	9.77 ± 0.46	9.77 ± 0.46	1.00^*∗*^

^a^P1: dentate point 1, P2: dentate point 2, P3: dentate point 3, P4: dentate point 4, EP1: edentulous point 1, EP2: edentulous point 2, EP3: edentulous point 3, and ^*∗*^nonstatistically significant *p* > 0.05.

**Table 2:** Bone thickness (mm) at 4 points of dentate sites on buccal and lingual sides according to the site (upper or lower jaw).

Points	*N*	Upper jaw		Lower jaw	
		Buccal	Lingual	Buccal	Lingual
P 1	80	1.14 ± 0.15	1.18 ± 0.18	1.16 ± 0.15	1.20 ± 0.19
P 2	80	1.28 ± 0.22	1.32 ± 0.24	1.30 ± 0.26	1.34 ± 0.27
P 3	80	1.79 ± 0.46	1.87 ± 0.54	1.81 ± 0.45	1.96 ± 0.61
P 4	80	3.17 ± 0.55	3.27 ± 0.61	3.35 ± 0.54	3.44 ± 0.59
*P* value		<0.001^*∗*^	<0.001^*∗*^	<0.001^*∗*^	<0.001^*∗*^

P: point, *N*: number, and ^*∗*^highly statistically significant.

**Table 3:** Comparison of bone width (mm) between upper and lower jaw of dentate sites at buccal and lingual sides according to the points.

Points	Buccal		*P* value	Lingual		*P* value
	Upper	Lower		Upper	Lower	
P 1	1.14 ± 0.15	1.16 ± 0.15	0.4	1.18 ± 0.18	1.20 ± 0.19	0.47
P 2	1.28 ± 0.22	1.30 ± 0.26	0.6	1.32 ± 0.24	1.34 ± 0.27	0.6
P 3	1.78 ± 0.46	1.81 ± 0.45	0.7	1.87 ± 0.54	1.96 ± 0.61	0.3
P 4	3.17 ± 0.55	3.35 ± 0.54	0.04^*∗*^	3.27 ± 0.61	3.44 ± 0.59	0.08

P: point and ^*∗*^statistically significant *P* < 0.05.

**Table 4:** Alveolar bone width (mm) of upper and lower jaws at edentulous sites.

Points	*N*	Upper	Lower	*P* value
EP 1	80	4.0 ± 0.47	4.09 ± 0.50	0.25
EP 2	80	7.32 ± 0.62	7.41 ± 0.47	0.31
EP 3	80	9.61 ± 0.75	9.75 ± 0.60	0.2
*P* value		<0.001^*∗*^	<0.001^*∗*^	

EP: edentulous point, *N*: number, and ^*∗*^highly statistically significant.

## Data Availability

The data used to support the findings of this study are available upon request.
